# An 8-week 24-form Tai Chi intervention on cognition in Chinese college students overusing short videos: a randomized controlled trial

**DOI:** 10.3389/fpsyg.2025.1517827

**Published:** 2025-09-02

**Authors:** Yu-fan Li, Chong-yao Xiao, Rong-xin Cai

**Affiliations:** ^1^School of Outdoor Sports, Guilin Tourism University, Guilin, China; ^2^College of Physical Education and Health, Guangxi Normal University, Guilin, China; ^3^School of Education and Health, Guilin Institute of Information Technology, Guilin, China; ^4^General Education College, Liming Vocational University, Quanzhou, China

**Keywords:** Tai Chi, cognition, college student, short video, sustained attention

## Abstract

**Introduction:**

This study evaluated the effect of an 8-week practice of 24 forms of Tai Chi on cognition in college students aged 18–21 years who were overexposed to short videos.

**Methods:**

A total of 1803 short video usage questionnaires were distributed at a college in Fujian Province, China, and 1790 valid questionnaires were collected. Sixty college students who scored in the top 20% on the questionnaire were randomly assigned to a Tai Chi experimental group (TCG) or an overuse control group (OCG), with 30 participants in each. The 32 students with the lowest 20% questionnaire scores were selected as the low-frequency-use control group (LCG). The TCG participated in 24-form Tai Chi training three times a week for 8 weeks. The OCG and LCG maintained their original habits without intervention. All groups were tested before, at the end of, and 2 months after training, using the digit string memory test, word delineation test, and three E-prime tests, which test memory, sustained attention, selective attention, and comprehensive attention.

**Result:**

There were no significant differences in the digital string memory test, the number cancellation test, and oddball paradigm results. In the Go/No-go experiment, accuracy showed no significant difference across groups before and after the intervention. Reaction times for TCG and OCG were significantly higher than LCG pre-intervention (*p* < 0.05). Post-intervention, TCG reaction times significantly decreased (*p* < 0.05), aligning with LCG and differing significantly from OCG. Two months post-experiment, reaction times were similar across all groups. In the Stroop test, no significant accuracy differences were found before and after the intervention. Pre-intervention, TCG and OCG reaction times were significantly longer than LCG (*p* < 0.05). Post-intervention, reaction times decreased for TCG and OCG, with TCG’s decline being more significant, aligning with LCG (*p* < 0.05). Two months post-experiment, reaction times became similar across all groups with no significant differences.

**Discussion:**

Overuse of short videos correlates with declines in sustained and comprehensive attention in college students. Tai Chi positively impacted sustained and comprehensive attention in students overusing short videos but had no significant effect on memory and selective attention. When Tai Chi training is discontinued, its positive effects gradually dissipate.

## 1 Introduction

Short-video apps have become a normal part of modern people’s lives. While short-video apps have many advantages, many people have reported that excessive use of short video applications has caused them to develop addictions, such as addiction to short videos, social media, and the Internet (Gao et al., 2017; Olasina and Kheswa, 2021). However, short video content differs from content on social network services (SNS) or the Internet in several key ways; specifically, short video content: (a) is simple and can be understood without a complex knowledge background; (b) tells very short stories that can be watched as fragments; and (c) involves more dramatic conflicts in a short period of time, without significant foreshadowing. These characteristics make short video addiction different from other similar addictions (e.g., video game and Internet addiction). Furthermore, short video addiction can develop quickly and without long-term engagement (Jianfeng et al., 2024). This leads to fundamental differences in how people develop their addiction. Short video addiction is defined as addictive behavior in which users use short video software in a dependent, inappropriate, or excessive manner (Ye et al., 2022). Many studies have pointed out that college students, in particular, are susceptible to Internet overuse (Davis, 2001). Given that college students are susceptible to Internet overuse, they are likely also vulnerable to short video overuse. According to past data, most college students watch short videos for more than 30 min, or even several hours per day. If college students choose to spend considerable time on short videos, their real-life activities, such as daily life, interpersonal communication, and learning skills, are greatly hampered. Among a sample of 908 Finnish adults, 20% believed that Internet and computer use impaired their cognitive abilities, particularly their memory and concentration (Näsi and Koivusilta, 2013). Emerging evidence suggests problematic short-video use involves multidimensional determinants, including individual traits (e.g., impulsivity), social-environmental factors (e.g., peer influence), and platform design features (e.g., autoplay, algorithmic recommendations) (Xiong et al., 2024). Concurrently, the interplay between flow states and media multitasking may exacerbate smartphone overuse and attentional fragmentation (Wickord and Quaiser-Pohl, 2025). These mechanisms provide a more comprehensive framework for understanding cognitive impacts of digital overuse.

Meditation training and physical activity are two effective training methods to promote attention (Posner et al., 2015; Tang and Posner, 2009). Meditation, especially mindfulness meditation, is a method widely used to enhance sustained attention (Posner et al., 2015). Mindfulness has been defined as “raising consciousness through the purpose, the current purpose and the expansion of experience without judgment” (Kabat-Zinn, 2003, p. 145). Mindfulness training can improve children’s self-regulation through top-down (controlled) and bottom-up (automatic) processes (Zelazo and Lyons, 2012). Meditation training can help individuals regulate their physiology and cerebral cortex, calming them, helping them reevaluate where their attention should be focused, helping them focus on certain targets, and allowing them to zone out other stimuli. Several studies have shown, using various different approaches, that physical activity can enhance attention (Vazou et al., 2016) and reduce addictive behavior (Roberts et al., 2012). According to a cross-sectional study, physical activity is directly related to enhanced cognitive function (Hamer and Chida, 2008). In practice, meditation and physical activity are the most commonly used interventions to enhance attention.

Tai Chi is a kind of physical exercise that combines physical activity and meditation. It connects action and concept. In the process of performing Tai Chi, not only is the body trained, but various cognitive abilities are also trained. Tai Chi is popular, especially in the older adult population, due to its smooth movements, low exercise intensity, and lack of need for equipment or a specific venue. Sports science research has revealed that Tai Chi can improve gait, physical fitness, and cardiovascular health, and that it is a safe and low-cost complementary therapy (Barnes, 2004; Birdee et al., 2009). Past psychological studies have shown that Tai Chi can improve older adults’ cognitive function and mental health (Lam et al., 2012; Man et al., 2010; Matthews and Williams, 2008). In recent years, increasing numbers of young people have also been exposed to Tai Chi. Studies have pointed out that young people’s participation in Tai Chi can improve their physical and mental health, mindfulness, mood, perceived stress, and sleep quality (Caldwell et al., 2016; Zheng et al., 2015). Thus, it is reasonable to believe that Tai Chi may play a positive role in improving the cognitive function of college students who overuse short videos.

Our study’s hypothesis is that 8 weeks of Tai Chi training will positively affect the cognitive abilities of college students who overuse short videos. Confirming or rejecting this hypothesis would provide some theoretical support for formulating strategies for improving college students’ physical health and cognitive abilities.

## 2 Materials and methods

### 2.1 Participants

This randomized controlled trial utilized the Short Video Internet Disorder Questionnaire (SIVD-Q) to screen 1,803 college students at a university in Fujian Province, China, with 1,790 valid responses collected. The Short Video Internet Disorder Questionnaire (SIVD-Q) is a 41-item self-report measure assessing six dimensions of problematic short-video use: Salience (cognitive preoccupation), Withdrawal (negative affect during abstinence), Loss of Control (unsuccessful attempts to reduce use), Mood Modification (emotional regulation through use), Health Problems (physical consequences), and Sleep Problems (nocturnal disruption) (Bai, 2024, p. 45). Items are rated on a 5-point Likert scale (1 = strongly disagree to 5 = strongly agree), with higher total scores (range: 41–205) indicating greater severity of addictive tendencies. Psychometric analyses demonstrated excellent reliability (Cronbach’s *α* = 0.96 for total scale; subscale *α* = 0.84–0.93; 2-week test–retest ICC = 0.85) and validity: confirmatory factor analysis supported the 6-factor structure (*χ*^2^/df = 3.61, CFI = 0.90, TLI = 0.90, RMSEA = 0.06), with strong subscale-total correlations (|*r*| ≥ 0.40) and convergent validity with established measures (IGDS9-SF: *r* = 0.67, *p* < 0.001; PHQ-9: *r* = 0.49, *p* < 0.001). The SIVD-Q shows discriminant validity through significant gender differences (female > male; *t*(817) = −4.46, *p* < 0.001, Cohen’s *d* = 0.32) and grade-level variations (sophomore > junior > freshman > senior; *F*(3,815) = 4.39, *p* = 0.005, *η*^2^ = 0.04), but no significant differences by only-child status (*t* = −0.68, *p* = 0.50). Participants were stratified based on SIVD-Q scores: the top 20*%* (*n* = 360) were classified as excessive short-video users, while the bottom 20% (*n* = 360) were designated as low-frequency users. From the excessive-use cohort, 60 students were randomly allocated to either the Tai Chi experimental group (TCG; *n* = 30) or the overuse control group (OCG; *n* = 30). An additional 32 students were randomly selected from the low-frequency group to form the low-frequency control group (LCG).

Sample size was determined *a priori* using GPower 3.1, with parameters set to detect a moderate effect size (*d* = 0.5, based on prior Tai Chi cognition studies), *α* = 0.05 (two-tailed), and power (1–*β*) = 0.80. The analysis indicated a minimum requirement of 25 participants per group; to account for attrition, we enrolled 30 per experimental group and 32 in LCG. During the intervention, two participants from TCG and one from OCG withdrew, resulting in final samples of TCG (*n* = 28), OCG (*n* = 29), and LCG (*n* = 32). Baseline demographic comparisons confirmed no significant differences in age or sex (*p* > 0.05). All participants provided informed consent and could withdraw unconditionally, per approval by the institutional ethics review board.

To ensure internal validity, we implemented rigorous controls for potential confounders. All participants maintained their baseline media consumption patterns, with weekly verification of screen time. The intervention group abstained from additional mind–body practices during the study period. Cognitive assessments were conducted by blinded evaluators to prevent measurement bias. These controls effectively isolated the experimental intervention’s effects while maintaining ecological validity.

### 2.2 Procedures

**Figure 1:**
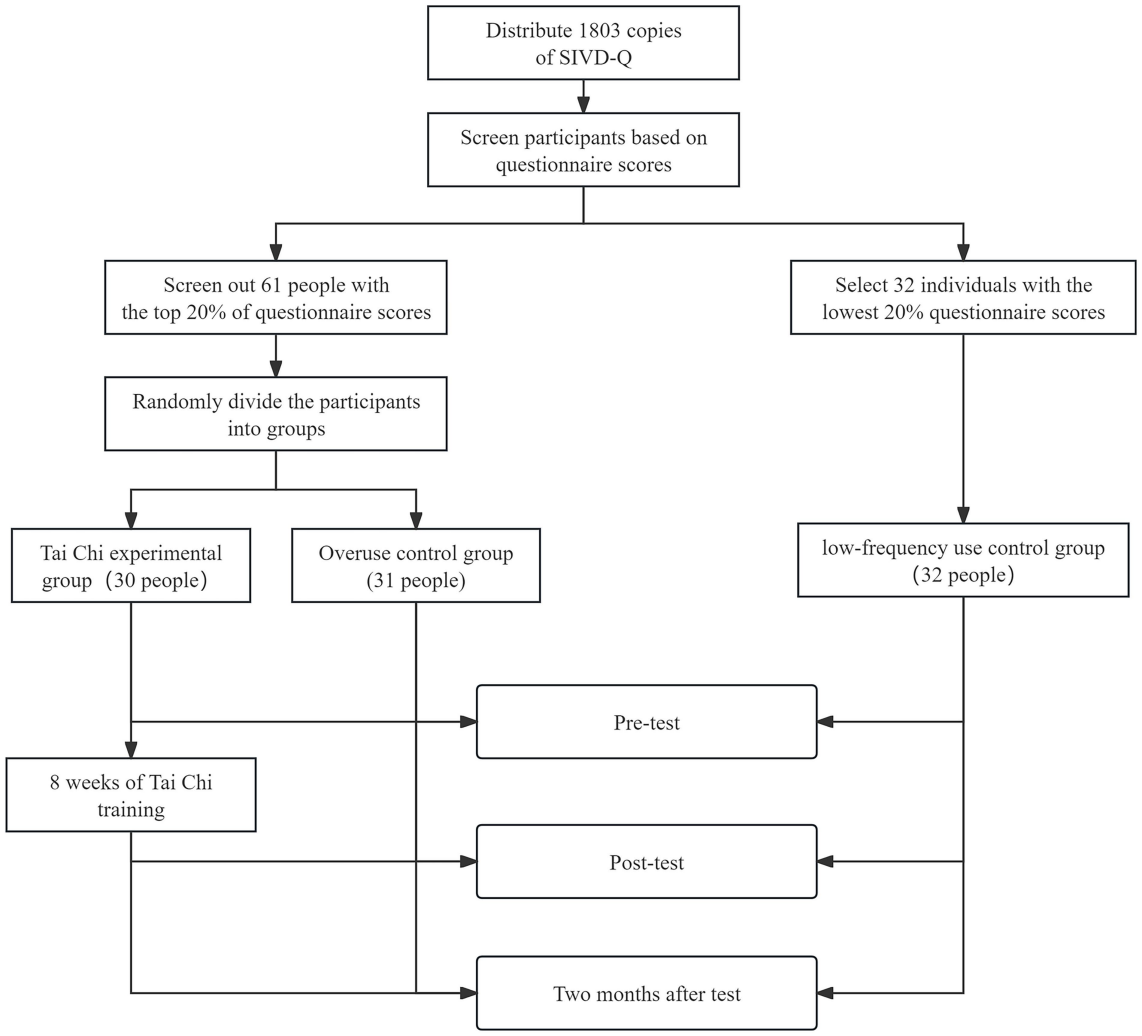
Data collection procedure.

The Tai Chi experimental group (TCG) completed an 8-week, 24-form Tai Chi program (3 sessions/week, 1 h/session) led by an experienced instructor. Each session included: 5-min warm-up, 10-min standing meditation, 40-min Tai Chi practice, and 5-min cool-down. Participants attended ≥85% sessions and passed skill assessments. To ensure internal validity, we: (1) maintained all participants’ baseline media consumption with weekly screen-time logs, (2) prohibited TCG from additional mind–body practices, and (3) implemented blinded cognitive assessments. Control groups (OCG, LCG) maintained regular lifestyles without intervention. Cognitive testing (digital string memory, number cancellation, E-Prime paradigms) was conducted at baseline, post-intervention, and 2-month follow-up, with SIVD-Q readministration at follow-up (see Figure 1).

### 2.3 Digital string memory experiment

Digital string memory experiments are typically used to study memory and to sustain attention. Before the experiment, participants were required to repeat a series of sequences composed of random numbers. Starting from three digits, as the difficulty increases, it can reach a maximum of over a dozen digits. All experiments were performed by the same person. After the participants completed the current question, the next question was one more number than the previous question. Until the participant could not repeat the number string, the test was repeated with the same difficulty. If the participant was unable to repeat the experiment, it was completed. This experiment used a random number length of participants as the evaluation indicator.

### 2.4 Number cancellation test

The number cancellation test (NCT) includes one short practice sheet and four test sheets for evaluating attention sustainability, which are reflected in the scores generated according to the accuracy of the test. Each sheet contained 200 digits with symbols below or above them. The targets are “9” digits with two symbols below, above, or on either side. Participants were asked to cross out targets with a slash and ignore targets placed subsequent to a “5” as quickly as possible, within 1 min for each sheet. The participants had a 10- to 20-s interval break between the two sheets. The final scores were calculated as the sum of the correct numbers of target digits for each sheet.

### 2.5 EPRIME cognitive experiment

EPRIME is a commonly used psychological experimental tool that is mainly used to test reaction time and accuracy rate, and has high flexibility and manipulability. This experiment comprised three types of experiments: Go/No-go, oddball, and Stroop. Before the formal test, each experimental paradigm had a trial phase to ensure that the participants understood the experimental rules. The experiment was conducted in a school computer laboratory. It uses the same computer model and installs the same version of EPRIME.

#### 2.5.1 Go/No-go paradigm

The Go/No-go paradigm (Benikos et al., 2013) was used to measure sustained attention (O'Connell et al., 2009). The participants are required to quickly press the space key when the screen presents “O.” If it does not operate, it does not answer the next question. When the screen presents “Q,” the participants do not need to do any operation, and the next question will automatically begin after 2 s. The experiment consisted of 150 questions. The probability of “O” appearing is 80%, and the probability of “Q” appearing is 20%. Accuracy and reaction time were used as indicators to evaluate sustained attention.

#### 2.5.2 Oddball paradigm

In this experiment, the participants were required to pay attention to random arrows presented on the screen and then determine whether the direction of the arrow that appeared immediately was consistent with the direction of the earlier arrow. They were asked to press the “F” key if the direction of the arrow was consistent and to press the “J” key if not. The experiment consisted of 150 questions. The probability of arrows moving in the same direction was 75% and the probability of arrows moving in inconsistent directions was 25%. This experiment studied selective attention and control capabilities through the consistency rate and reaction at different times when the research direction was consistent and different.

#### 2.5.3 Stroop test

In this experiment, the participants determine whether the meaning of the Chinese text “red, yellow, blue, and green” on the screen is consistent with the colors listed. If the word “blue” was written in blue, they were asked to press the “F” key. If “blue” was written in green, they were asked to press the “J” key. The color and text combinations were random and the probability of matching was 25%, while the probability of inconsistency was 75%. The experiment had a total of 150 questions. This experiment comprehensively studied accuracy and reactions under different circumstances.

### 2.6 Statistical analysis

Data analysis was performed using SPSS software. Means and standard deviations were calculated for all the variables. Missing data were imputed using mean substitution, and data points greater than three standard deviations from the mean were excluded. Normality was evaluated by visual inspection and the Shapiro–Wilk test, and non-normal measures were log-transformed prior to correlation and inferential analyses. Data normality was confirmed using the Kolmogorov–Smirnov test (*p* > 0.05), and homogeneity of variances was assessed using the Levene test. A two-way repeated measures ANOVA was used to examine the main effects and interactions of time (pre-test vs. post-test vs. 2-months-post-test) and group (TCG vs. OCG vs. LCG) on the selected outcomes. Statistical analyses confirmed that all variables met the assumptions of normality (Kolmogorov–Smirnov test: all *p* > 0.05) and homogeneity of variance (Levene’s test: all *p* > 0.05), satisfying the prerequisites for parametric testing. Following the ANOVA, systematic *post hoc* paired-sample *t*-tests were conducted to assess within-group changes over time for each measured variable.

## 3 Results

**Figure 2:**
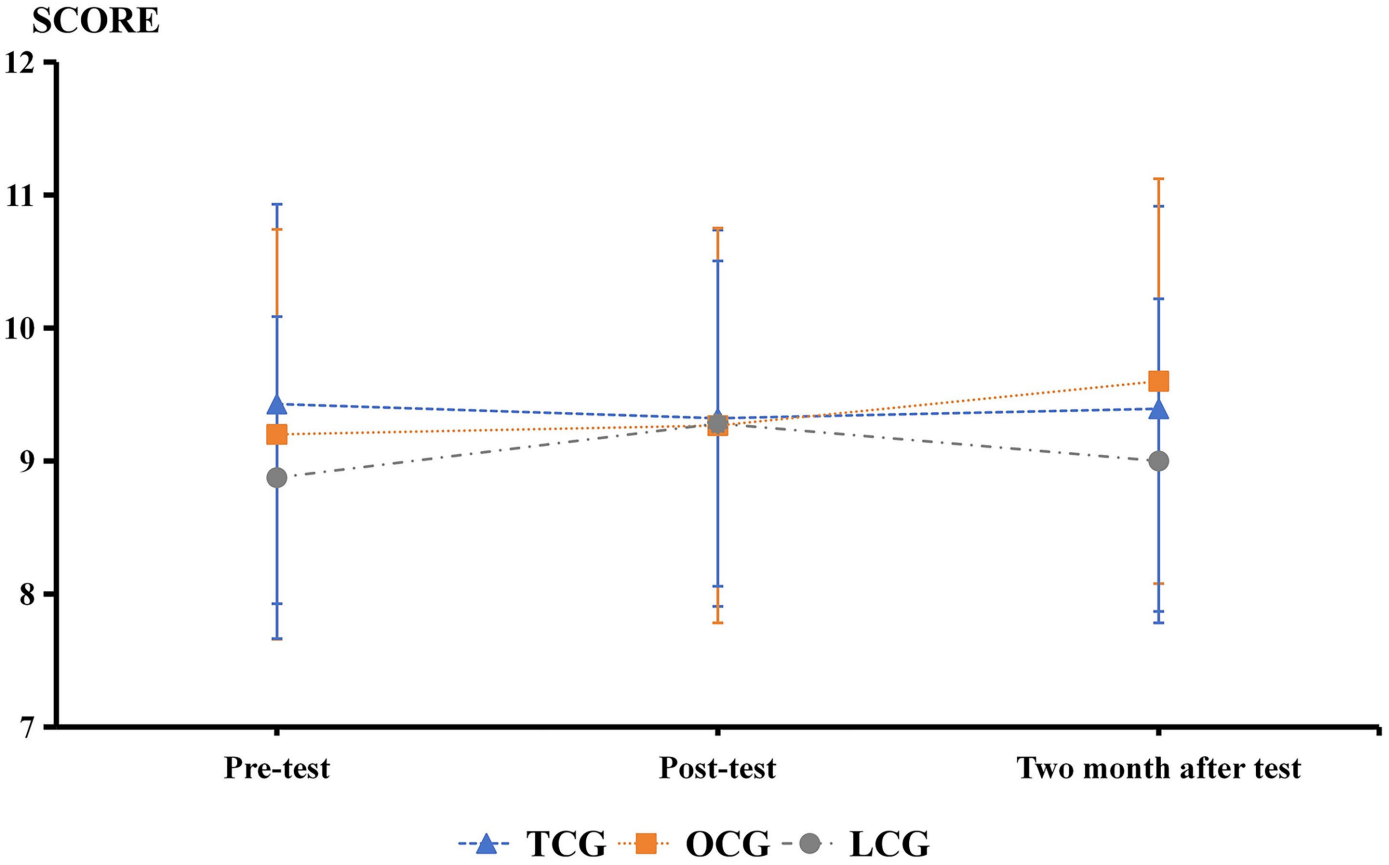
Digital string memory experiment results.

**Figure 3:**
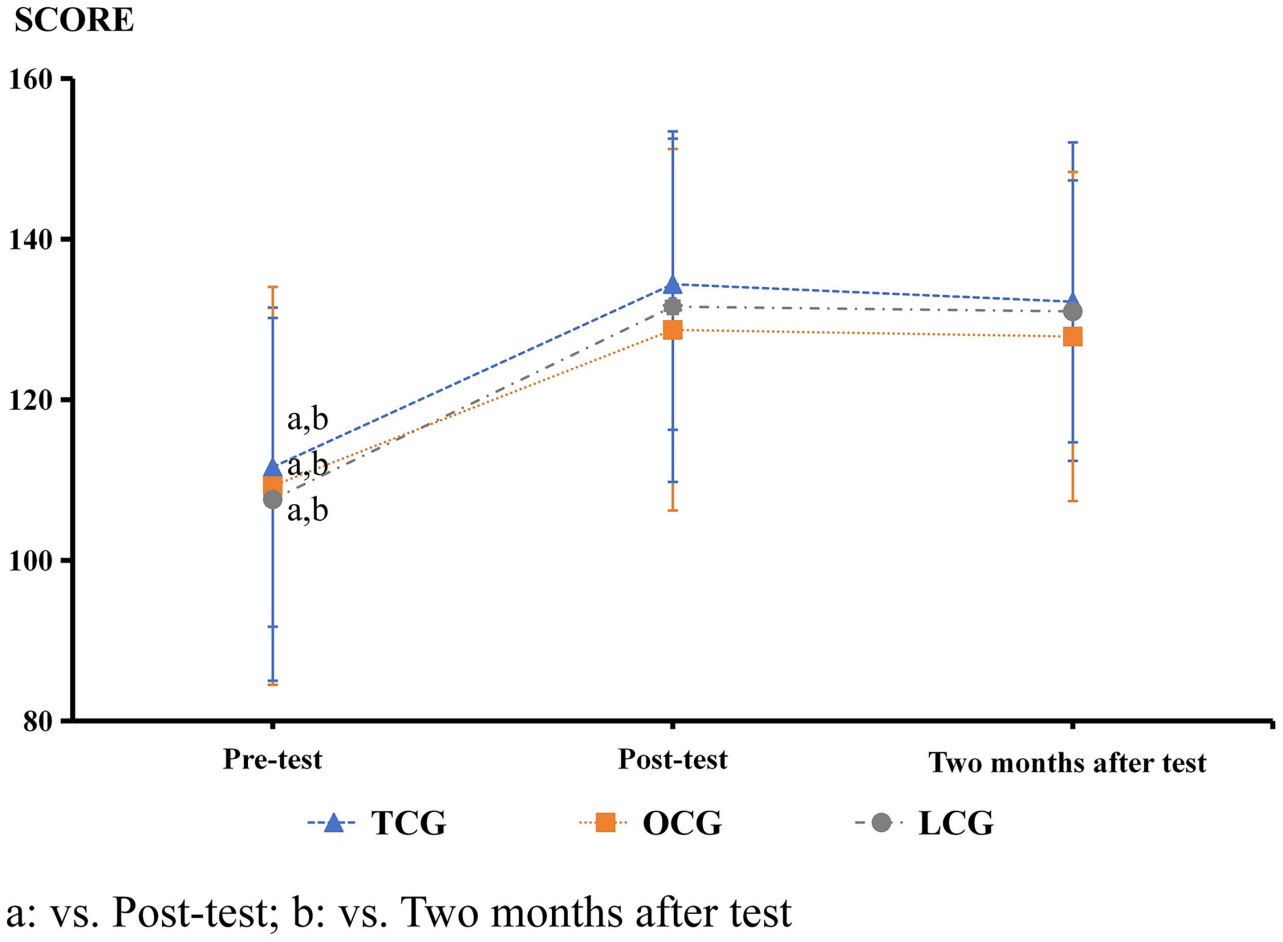
Number cancellation test results.

**Figure 4:**
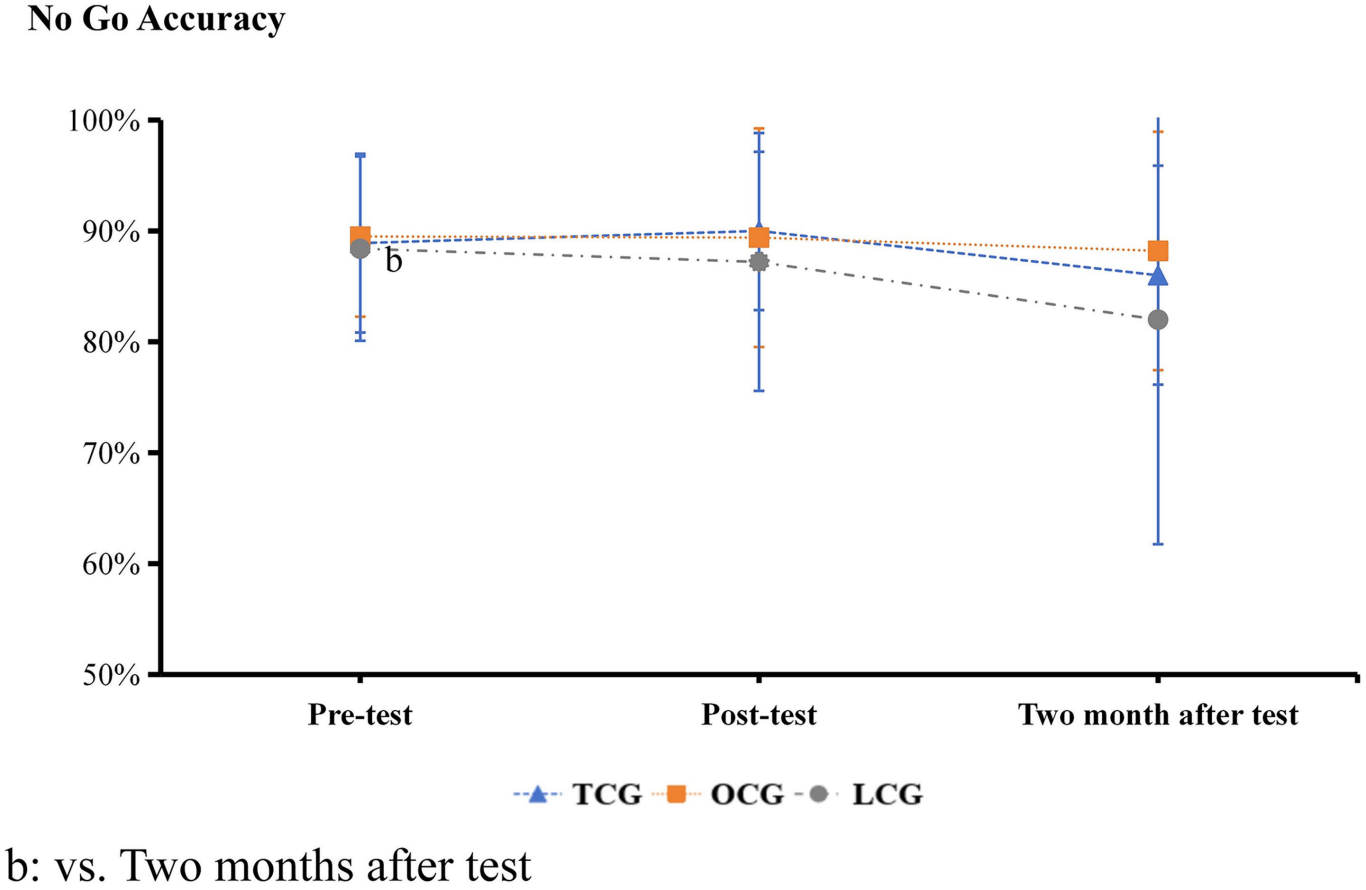
The Go/No-go paradigm accuracy results.

**Figure 5:**
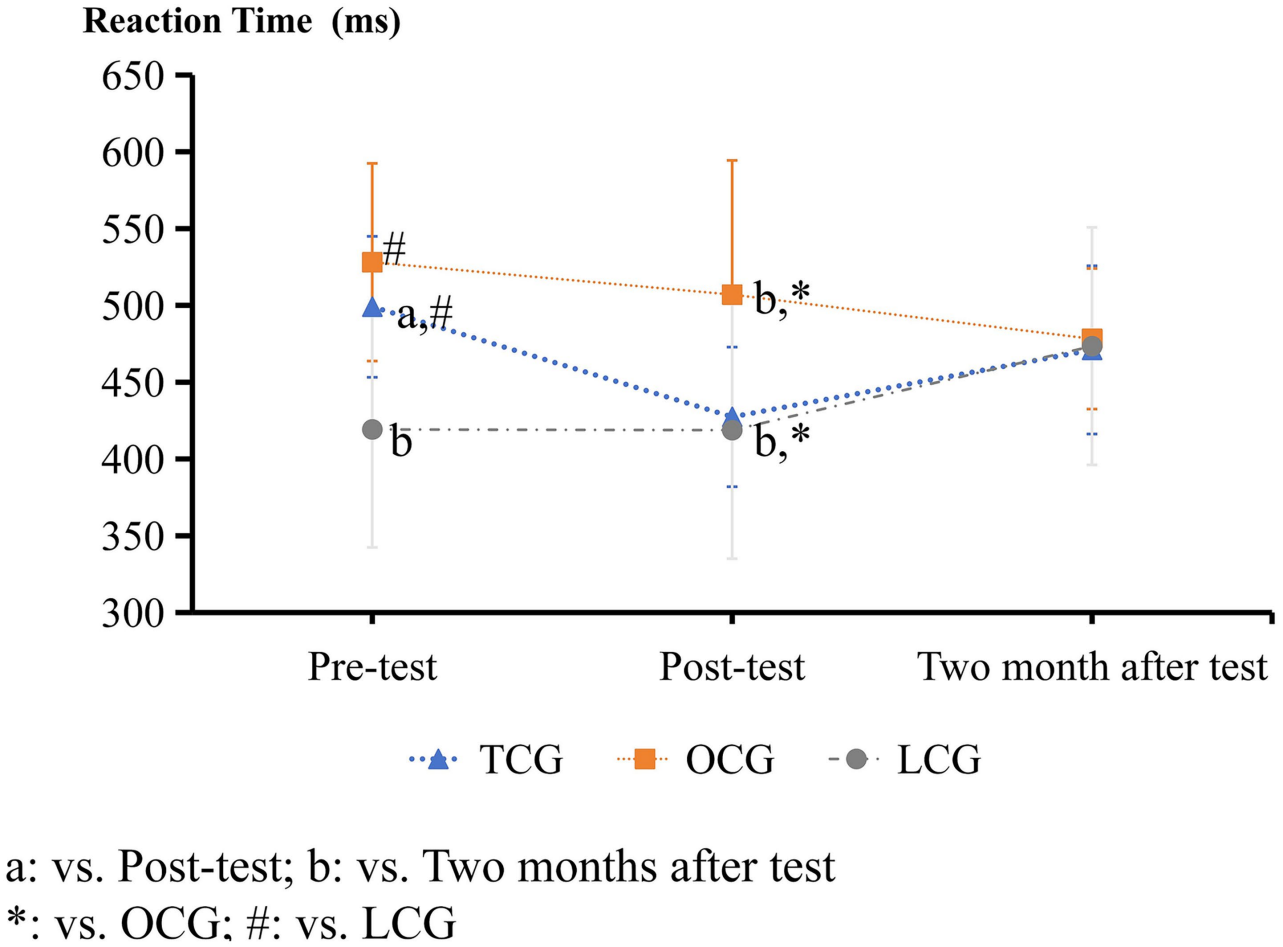
The Go/No-go paradigm reaction time results.

**Figure 6:**
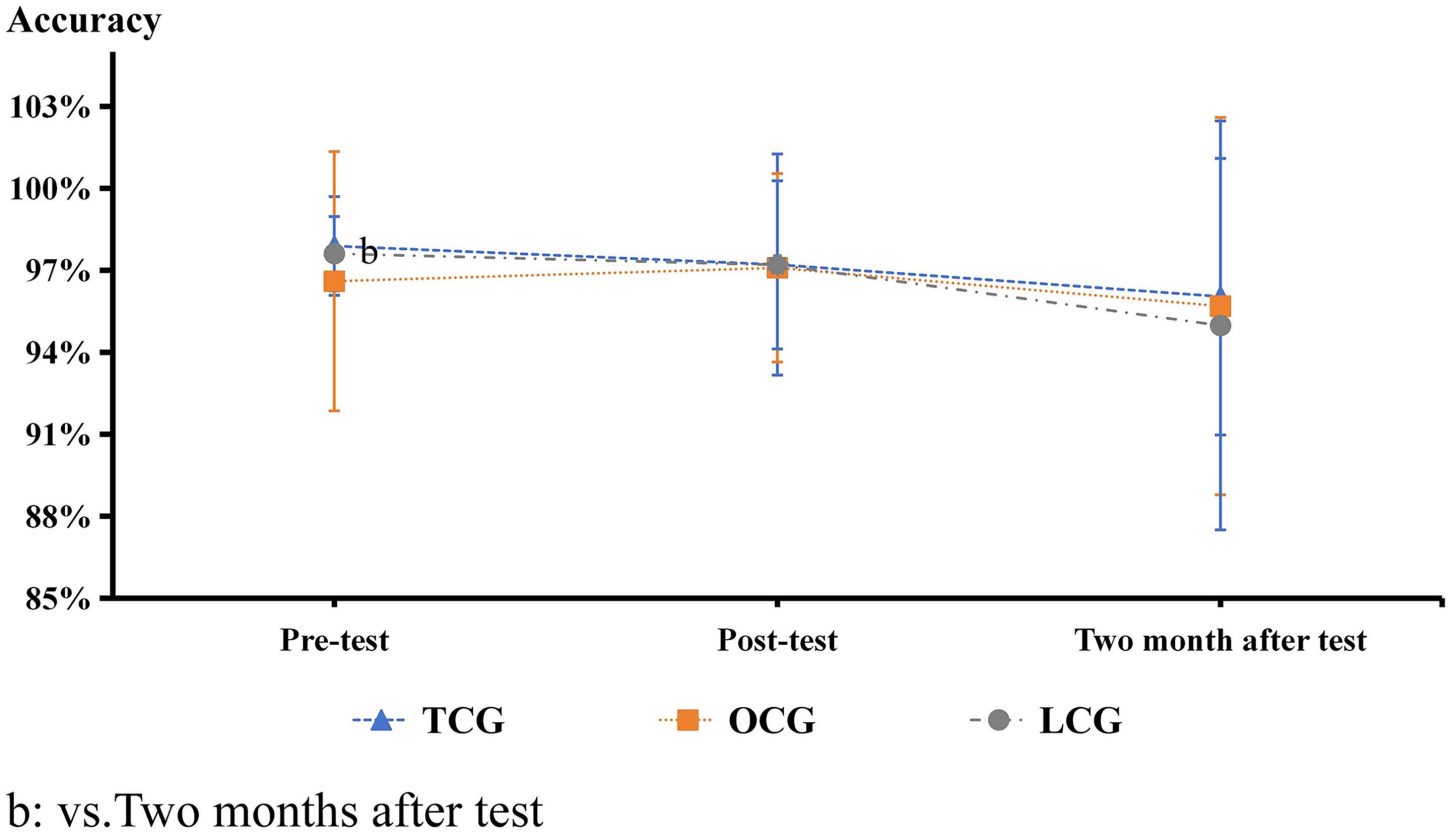
Oddball paradigm accuracy results.

**Figure 7:**
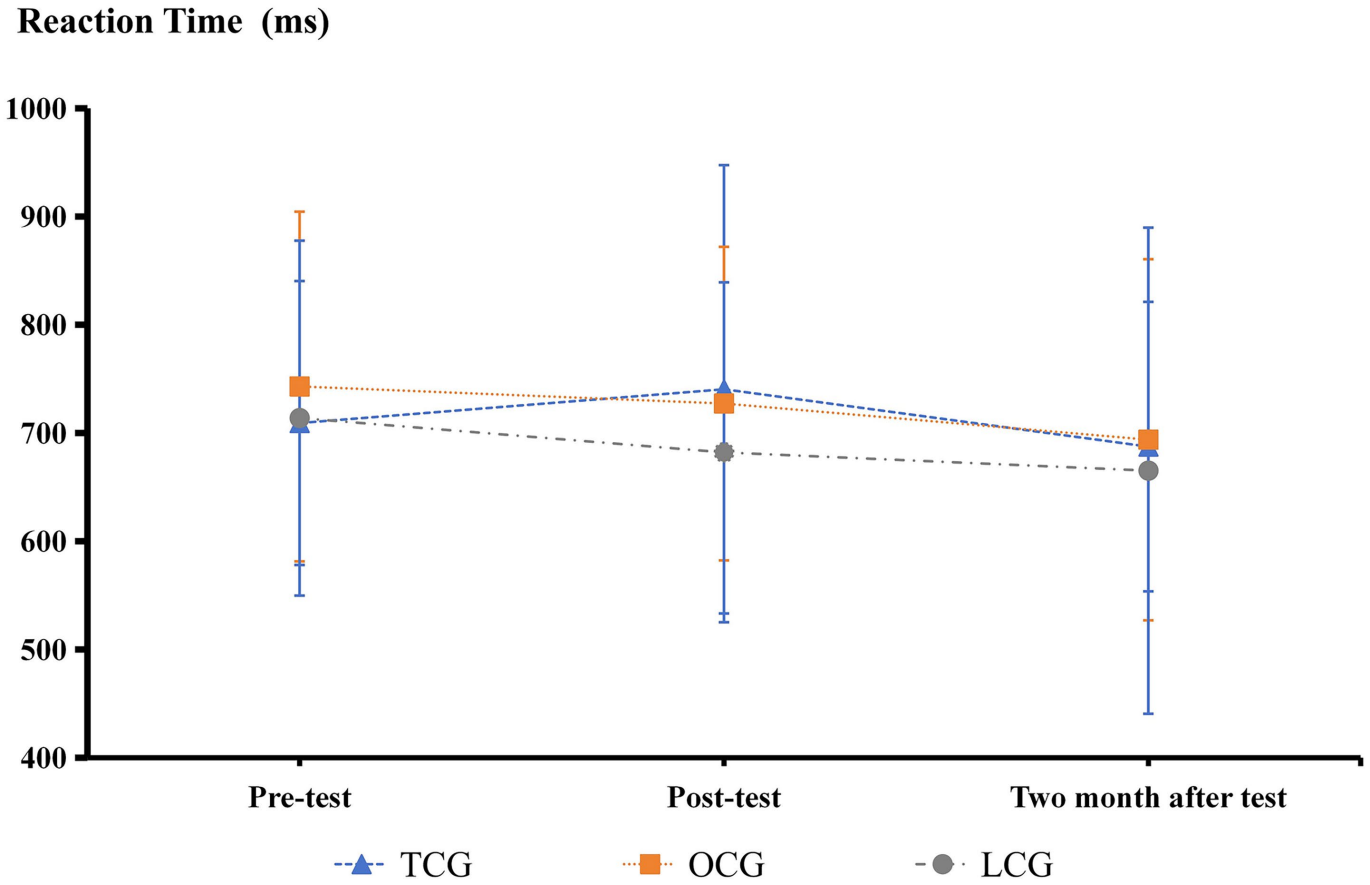
Oddball paradigm reaction time results.

**Figure 8:**
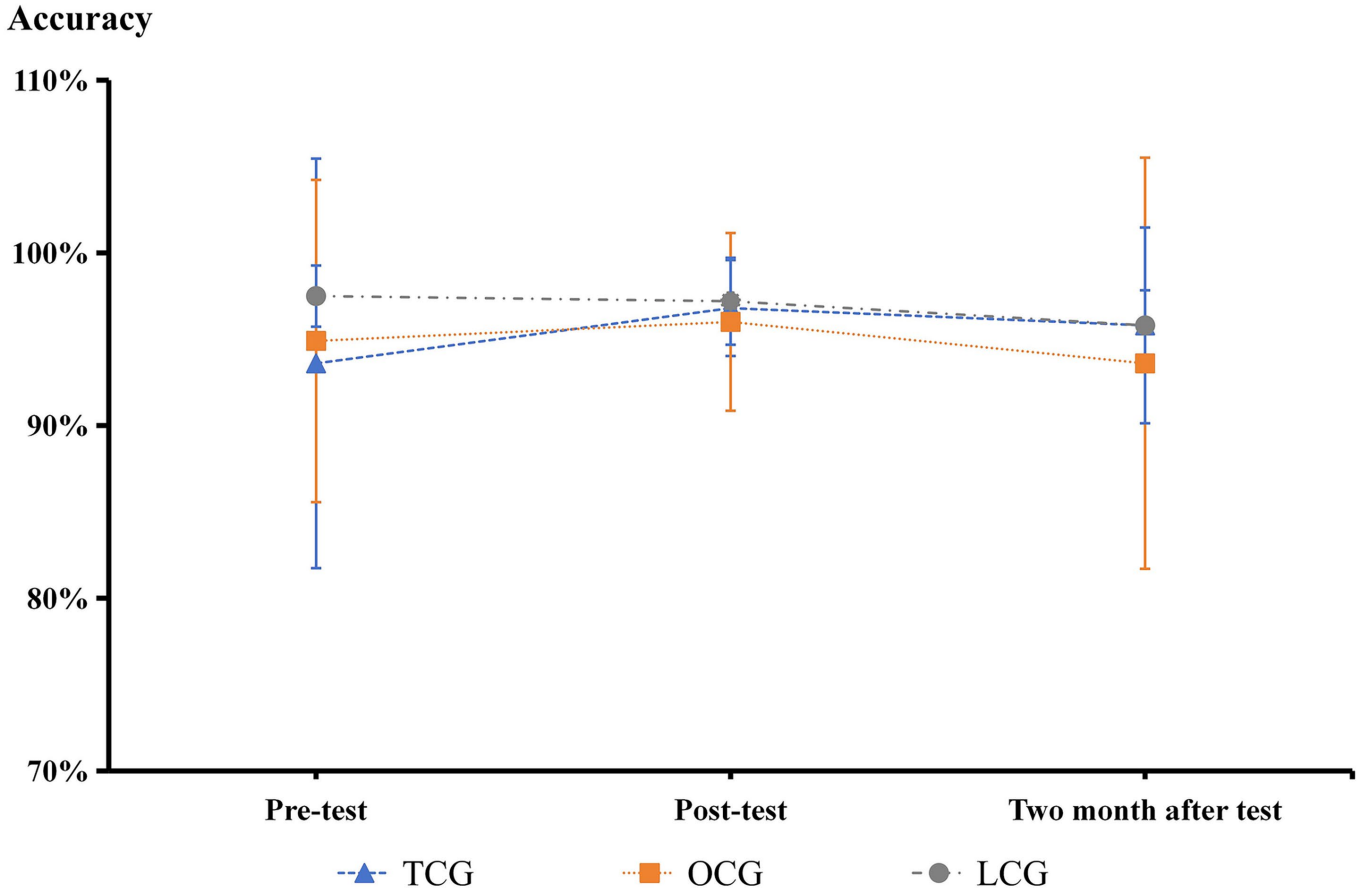
Stroop paradigm accuracy results.

**Figure 9:**
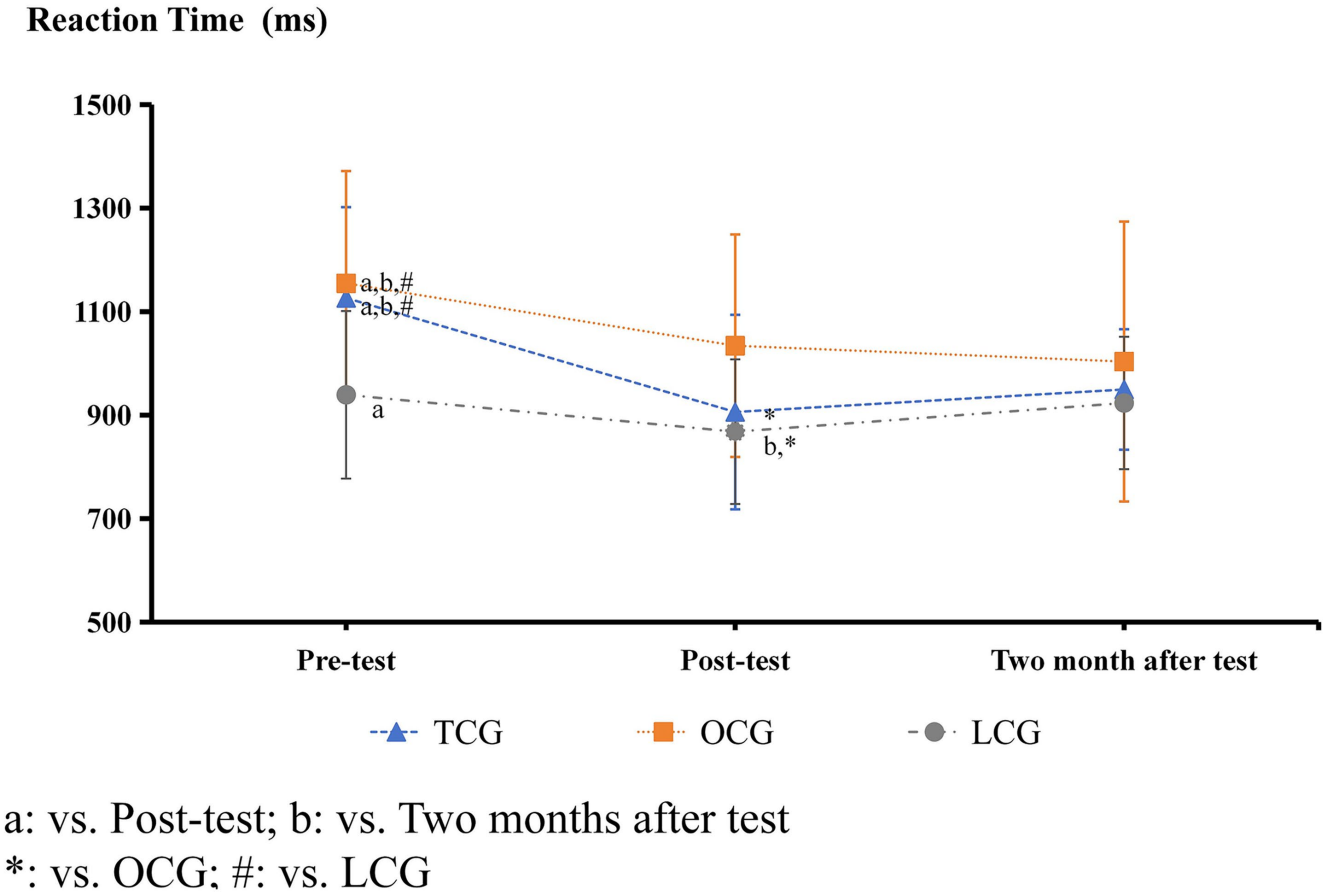
Stroop paradigm reaction time results.

Inter-correlations between paradigms ranged from *r* = 0.32 to 0.41, indicating assessment of related but distinct cognitive domains. The results presented in Table 1 and Figures 2–9 show significant interactions between group and time for several tests, and there were no significant differences in the results of the digital string memory experiment, the number cancellation test, and oddball paradigm.

**Table 1:** Comprehensive outcomes of three groups at three assessment phases.

Experimental paradigm	Indicators	Groups	Pre-test (Mean ± SD)	Post-test (Mean ± SD)	2 months after test (Mean ± SD)	Statistics (*F*, *p*, *η*^2^)
GO-no-go paradigm	Go reaction time (ms)	LCG	419.22 ± 76.72^b^	418.75 ± 83.63^b,#^	473.44 ± 77.34	*F* = 3.06, *p* = 0.050, *η*^2^ = 0.034
		OCG	528.11 ± 64.38^#^	507 ± 87.5	478.19 ± 45.76	
		TCG	499.13 ± 45.91^a,#^	427.4 ± 45.46^b,#^	471.01 ± 54.79	
	Nogo accuracy (%)	LCG	88% ± 8%^b^	87% ± 12%	82% ± 2%	*F* = 0.39, *p* = 0.816, *η*^2^ = 0.009
		OCG	90% ± 7%	89% ± 10%	88 ± 11%	
		TCG	89 ± 8%	90 ± 7%	0.86% ± 10%	
Oddball paradigm	Accuracy (%)	LCG	98% ± 1%^b^	97% ± 3%	95% ± 7%	*F* = 1.73, *p* = 0.180, *η*^2^ = 0.020
		OCG	97 ± 5%	97% ± 3%	96% ± 7%	
		TCG	98 ± 2%	97 ± 4%	96 ± 5%	
	Reaction time (ms)	LCG	713.81 ± 164.03	682.04 ± 157.04	665.19 ± 224.55	*F* = 0.59, *p* = 0.669, *η*^2^ = 0.013
		OCG	742.99 ± 161.53	727.21 ± 144.88	693.81 ± 166.9	
		TCG	709.19 ± 131.08	740.4 ± 207.24	687.37 ± 133.75	
Stroop test	Accuracy (%)	LCG	98% ± 2%	97% ± 3%	96% ± 6%	*F* = 1.04, *p* = 0.389, *η*^2^ = 0.023
		OCG	95 ± 9%	96% ± 5%	94% ± 12%	
		TCG	94% ± 12%	97% ± 3%	96 ± 2%	
	Reaction time (ms)	LCG	939.28 ± 161.75^a^	867.88 ± 139.97^b,*^	923.41 ± 127.8	*F* = 9.39, *p* < 0.001, *η*^2^ = 0.178
		OCG	1154.68 ± 216.68^a,b,#^	1034.12 ± 214.81	1003.44 ± 270.55	
		TCG	1126.33 ± 175.6^a,b,#^	906.01 ± 187.91^*^	949.57 ± 116.61	
Number cancellation test	(Score)	LCG	8.88 ± 1.21	9.28 ± 1.22	9 ± 1.22	*F* = 0.82, *p* = 0.442, *η*^2^ = 0.009
		OCG	9.2 ± 1.54	9.27 ± 1.48	9.6 ± 1.52	
		TCG	9.43 ± 1.5	9.32 ± 1.42	9.39 ± 1.52	
Digital string memory experiment	(Score)	LCG	107.59 ± 22.59^a,b^	131.59 ± 21.83	131 ± 16.32	*F* = 58.23, *p* < 0.001, *η*^2^ = 0.401
		OCG	109.27 ± 24.78^a,b^	128.7 ± 22.51	127.87 ± 20.51	
		TCG	111.61 ± 19.88^a,b^	134.39 ± 18.14	132.21 ± 19.82	

a: vs. Post-test; b: vs. 2 months after test; ^*^: vs. OCG; ^#^: vs. LCG.

In the Go/No-go experiment, there were no significant differences in accuracy between the three groups before and after the intervention (Figure 4). The reaction times of the TCG and OCG were significantly higher than those of the LCG before the intervention (*p* < 0.05). The post-paired *t*-test results of the TCG showed that the reaction time after the intervention was significantly decreased (*p* < 0.05), close to that of the LCG, which was significantly lower than that of the OCG. After 2 months, the reaction times of the three groups were essentially the same (Figure 5).

In the Stroop experiment, there were no significant differences in accuracy between the three groups before and after the intervention (Figure 8). Before the intervention, the reaction times of the TCG and OCG were significantly longer than those of the LCG (*p* < 0.05). After the intervention, the reaction times of the TCG and OCG decreased, but the decline in the TCG was more significant, close to that of the LCG, and there was a significant difference from that before the intervention (*p* < 0.05). After 2 months of the test experiment, the reaction times of the three groups became very similar, and there were no significant differences (Figure 9).

## 4 Discussion

University is an important stage of learning in individuals’ lives. Sustained attention strongly predicts academic performance (Chen and Wang, 2018). Sustained attention not only helps maintain the concentration needed for learning, but is also important for solving complex problems. The processes of strengthening the body through a combination of meditation and sports activities have been widely recognized; however, these activities’ impact on cognition has been comparatively less researched. Therefore, this study aimed to examine the effects of Tai Chi on the sustained attention of college students who overuse short videos. An important finding of this study is that the 8-week Tai Chi training significantly improved the sustained and comprehensive attention of college students who overused short videos. However, the effects on memory and selective attention were not significant. After 8 weeks post-training, Tai Chi’s effect on sustained and comprehensive attention dissipated and was no longer significant across the groups. The observed attention improvements may reflect Tai Chi’s capacity to modulate flow states. As Wickord and Quaiser-Pohl (2025) demonstrate, positive flow experiences can counteract attention dispersion from media multitasking—paralleling Tai Chi’s attention-enhancing mechanisms through its ‘dynamic-static integration’. Furthermore, Xiong et al.'s (2024) multidimensional model suggests future interventions should account for individual differences and platform characteristics as potential moderators.

In digital string memory experiments, the differences among the three groups were not significant before the experiment, indicating that the overuse of short videos did not significantly affect college students’ memory. The scores of the three groups after Tai Chi training are not significant, indicating that Tai Chi has no significant effect on the memory of college students. A possible reason for this is that college students’ brains are still developing, and their memory naturally improves during this period. Generally, scores on the digital string memory test are 7 ± 2 (Miller, 1956; Cowan, 2001). This study’s participants (every group) scored in a similar range. Previous studies have indicated that daily moderate-to-vigorous physical activity positively affects human memory (Bugg et al., 2006; Candela et al., 2015; Rebok and Plude, 2001), and attention (Felez-Nobrega et al., 2017). The exercise intensity of Tai Chi is low, and its effect on memory is not significant.

In the NCT, the scores of the three groups before the experiment were similar and the scores of the three groups increased in the second and third tests, indicating that this test had an obvious practice effect. With increasing proficiency, the performance gradually improved. The effect of practicing Tai Chi on this test was not significant.

In the Go/No-go paradigm, the accuracy rate of the three groups was high (close to 100%), but the scores of the three groups were not significant, which shows that the participants’ attitude was serious, and they gave priority to ensuring the accuracy rate. This also proves that the task has moderate difficulty for college students. If the experiment is too difficult, the correct response is difficult to identify, which reduces the accuracy rate. Reaction times in the TCG and OCG groups were significantly longer than those in the LCG group. This indicates that the overuse of short videos has a negative effect on sustained attention. Previous research suggests that action video games negatively affect sustained attention (Trisolini et al., 2018). However, when using short video applications, attention usually peaks in the first few seconds and then begins to decline. It is difficult to concentrate when continuously focusing on something, particularly something that is not interesting. Usually, teenagers handle multiple forms of media simultaneously, with a particular focus on the effect of media multitasking on sustained attention. Sustained attention can be divided into vigilance (detecting the appearance of a stimulus) and concentration (focusing on a stimulus or activity while filtering irrelevant distractors) (Sarter et al., 2001). Chen et al. (2023) found that addicted users reported less interest, centration, and more distractions and exhibited more fixation counts and shorter average fixation duration while watching short-form videos than non-addicted users. After Tai Chi training, the results of the TCG were improved to be comparable to those of the LCG, and the performance of the OCG was basically unchanged. This shows that Tai Chi training sustains the attention of college students who overuse short videos. A possible reason is that Tai Chi training pays attention to the “combination of dynamic and static.” The rhythm was not fixed during the Tai Chi training, unlike dance or gymnastics, in which the beats are fixed. Tai Chi’s rhythm is based on its own feelings, and the trainee needs to continue to pay attention to his/her own feelings, not only to remember the actions but also to grasp the rhythm. During the training process, sustained attention improved.

In the oddball test, the reaction time and accuracy of the three groups were not significantly different before the Tai Chi training. One possible reason for this is that attention is highly concentrated when viewing short videos. It can be switched over within a short time if it is not of interest. Selective attention is not involved. The scores of the three groups after intervention were not significantly different. Compared to the Go/No-go experiment, participants were required to respond to two situations in this experiment and interact with the outside world. Tai Chi is not a sport that emphasizes interaction with the external world. Instead, it focuses on one’s own feelings and internalization. Therefore, improvement in selective attention was not significant. According to previous studies, competitive confrontation projects, such as tennis and badminton, improve selective attention. Zhang et al. (2023) pointed out that acute moderate-intensity aerobic exercise significantly enhances speed accuracy and visual reaction time. As the exercise intensity of Tai Chi was relatively low, regardless of the type or intensity of exercise, the impact of Tai Chi on selective attention was not significant.

In the Stroop test, while accuracy rates remained consistently high across groups, the Tai Chi group demonstrated significantly reduced reaction times post-intervention, reaching levels comparable to the low-frequency users. These behavioral improvements may reflect three neurocognitive mechanisms: (a) enhanced conflict monitoring through anterior cingulate cortex engagement developed during Tai Chi’s meditative components (Posner et al., 2015), (b) strengthened prefrontal-striatal connectivity from the complex motor sequencing inherent in 24-form practice (Zheng et al., 2015), and (c) stress-mediated freeing of prefrontal resources via cortisol reduction (Caldwell et al., 2016). These mechanisms collectively explain the observed reduction in interference effects, suggesting that Tai Chi’s unique combination of movement and meditation trains both the capacity to maintain focused attention during prolonged tasks and the ability to efficiently resolve cognitive conflicts—precisely the demands challenged by the Stroop paradigm.

After Tai Chi training, 8 weeks post-intervention, the three groups completed the SIVD-Q. It was found that the scores of short video usage changed greatly, and that overuse of short videos was not a long and stable state, but rather changes with the state of life. The 8 weeks after the intervention were the Chinese winter vacations and the Spring Festival. During this holiday, the main entertainment method for many college students returning home was short videos, so their SIVD-Q scores changed to varying degrees. The grouping of the questionnaires was no longer reliable. The participants did not partake in Tai Chi training during the winter vacation, and the results obtained from the experiment were not significant. Post-training benefits exhibit domain-specific decay patterns, with motor skills showing persistent retention but cognitive gains like sustained attention requiring ongoing practice to maintain (Yang et al., 2024). The current findings demonstrate this dichotomy, as Tai Chi’s attention-enhancing effects diminished following training cessation despite the likely retention of movement sequences.

Several study limitations warrant consideration: First, the moderate inter-correlations between cognitive paradigms (*r* = 0.32–0.41) suggest our measures captured distinct attentional components, highlighting the need for multi-method validation in future research. Second, our extreme-groups design (selecting top/bottom 20% on SIVD-Q) enhanced group contrasts but may introduce regression to the mean and selection bias. While we controlled for key demographics, unmeasured individual differences could influence results. Third, the 8-week intervention period may be insufficient for lasting neural changes, consistent with prior Tai Chi studies (Zheng et al., 2015). Longer-term follow-ups are needed. Fourth, despite controlling major confounders (media use, exercise), unassessed factors (e.g., academic stress, sleep) may affect outcomes. Fifth, our design did not quantify platform-specific features (e.g., notification frequency, content types) that may interact with cognitive traits per Xiong et al.'s (2024) framework. Future studies should incorporate granular platform metrics. Finally, the Chinese college student sample limits generalizability, requiring cross-cultural replications.

## 5 Conclusion

This study revealed two key findings: (1) cross-sectional analyses showed short-video overuse correlated with poorer sustained attention (Go/No-go: *p* < 0.05, *d* = 0.42; Stroop: *p* < 0.05, *d* = 0.38), though causal direction remains unclear given the observational design; (2) the randomized trial demonstrated Tai Chi significantly improved sustained attention in overusers (ΔRT = −26 ms, *p* < 0.05, *d* = 0.49), with effects specific to attention domains and diminishing post-intervention. While supporting prior associations between media use and attention (Chen et al., 2023), these results extend the literature by showing Tai Chi’s potential as an intervention. The transient benefits suggest sustained practice may be needed, consistent with neuroplasticity models (Posner et al., 2015). Future work should examine causal pathways and optimal training durations.

## Data Availability

The original contributions presented in the study are included in the article/Supplementary material, further inquiries can be directed to the corresponding author.
